# A Maize *ZmAT6* Gene Confers Aluminum Tolerance *via* Reactive Oxygen Species Scavenging

**DOI:** 10.3389/fpls.2020.01016

**Published:** 2020-07-09

**Authors:** Hanmei Du, Ying Huang, Min Qu, Yihong Li, Xiaoqi Hu, Wei Yang, Hongjie Li, Wenzhu He, Jianzhou Ding, Chan Liu, Shibin Gao, Moju Cao, Yanli Lu, Suzhi Zhang

**Affiliations:** ^1^ Key Laboratory of Biology and Genetic Improvement of Maize in Southwest China of Agricultural Department, Ministry of Agriculture, Maize Research Institute, Sichuan Agricultural University, Chengdu, China; ^2^ Crop Research Institute, Sichuan Academy of Agricultural Sciences, Chengdu, China

**Keywords:** aluminum toxicity, *ZmAT6*, maize, reactive oxygen species, antioxidant

## Abstract

Aluminum (Al) toxicity is the primary limiting factor that affects crop yields in acid soil. However, the genes that contribute to the Al tolerance process in maize are still poorly understood. Previous studies have predicted that ZmAT6 is a novel protein which could be upregulated under Al stress condition. Here, we found that *ZmAT6* is expressed in many tissues and organs and can be dramatically induced by Al in both the roots and shoots but particularly in the shoots. The overexpression of *ZmAT6* in maize and *Arabidopsis* plants increased their root growth and reduced the accumulation of Al, suggesting the contribution of *ZmAT6* to Al tolerance. Moreover, the *ZmAT6* transgenic maize plants had lower contents of malondialdehyde and reactive oxygen species (ROS), but much higher proline content and even lower Evans blue absorption in the roots compared with the wild type. Furthermore, the activity of several enzymes of the antioxidant system, such as peroxidase (POD), superoxide dismutase (SOD), catalase (CAT), and ascorbate peroxidase (APX), increased in *ZmAT6* transgenic maize plants, particularly SOD. Consistently, the expression of *ZmSOD* in transgenic maize was predominant upregulated by Al stress. Taken together, these findings revealed that *ZmAT6* could at least partially confer enhanced tolerance to Al toxicity by scavenging ROS in maize.

## Introduction

Acidic soils refer to soil with pH ≤ 5.5, occupying almost 30% of the arable soil and 50% of the potential cultivated land (von Uexküll and Mutert, 1995). Aluminum (Al), the third abundant element in the earth’s crust, can be converted into soluble and toxic Al^3+^ which is the major limiting factor for plants’ growth in acidic soil (Kochian et al., 2004). Aluminum toxicity could rapidly inhibit the elongation of the root system even in a micromolar concentration, then affect the absorption of water and nutrients, and eventually resulting in the decline of crop yield (Kinraide, 1990; Ma and Furukawa, 2003; Kochian et al., 2004). Improving the physiological and genetic tolerance to Al in crops has long been a challenging problem for researchers.

To cope with Al stress, plants have evolved a series of aluminum resistant mechanisms. The primary Al-tolerance mechanisms in plants refer to the exclusion tolerance and internal tolerance (Kochian, 1995; Ma et al., 1997; Ma, 2000; Daspute et al., 2017). The common feature of the Al exclusion mechanisms is to prevent Al from entering the root apex by the excretion of detoxified organic acids (OAs) and Pi ligands to the apoplast or rhizosphere. The internal tolerance mechanism refers to the sequestration of Al into vacuoles and its detoxification by chelation (Ma, 2000; Zheng and Yang, 2005). Both mechanisms are controlled by the expression of a series of genes. In recent decades, many genes related to Al tolerance have been identified, such as organic acid transporter genes (*ALMTs* and *MATEs*) (Hoekenga et al., 2006; Furukawa et al., 2007; Liu et al., 2013), antioxidative stress-related genes (Wu et al., 2017), also including those encoding aluminum transporter (Nramps and ABC transporter, aquaporins) (Huang et al., 2012; Li et al., 2014; Kochian et al., 2015; Lu et al., 2017; Wang et al., 2017), enzymes related to cell wall polysaccharide metabolism (XTHs) (Zhu et al., 2012; Zhu et al., 2014), and transcription factor (STOP, WRKY, ASR, NAC) (Iuchi et al., 2007; Ding et al., 2013; Arenhart et al., 2016; Li et al., 2018; Lou et al., 2019).

Previous studies have reported that one of an important component of the plant’s reaction to toxic levels of Al is oxidative stress, because Al^3+^ can induce the increase of active reactive oxygen species (ROS) and lipid peroxidase related enzyme activities in plants such as soybean, Arabidopsis, wheat, and maize (Cakmak and Horst, 1991; Keith et al., 1998; Giannakoula et al., 2010; Xu et al., 2012; Sun et al., 2017). In other words, the exposure of plants to an excessive amount of Al usually leads to the overproduction of ROS (Yamamoto et al., 2001; Exley, 2004) and lipid peroxidation, resulting in dysfunctional organelles, serious cell damage, and even cell death (Yamamoto et al., 2003; Šimonovičová et al., 2004; Kochian et al., 2005). The major source of ROS in Al stressed plants is the activated plasma membrane NAPDH oxidase which can lead to the production of O_2_^·−^ and H_2_O_2_ (Sagi and Fluhr, 2001). Al^3+^ can quickly cross the plasma membrane and activate the Fenton reaction in the cytoplasm which increases the content of ROS in cells (Taylor et al., 2000).

To protect the plants from Al-triggered oxidative stress, they have evolved two defense ways, including enzyme-catalyzed antioxidant system and non-enzymatic system, to decrease ROS production, detoxify ROS, and stimulate the recovery from ROS-induced damages (Ahmad et al., 2010; Chowra et al., 2017; Daspute et al., 2017). The enzyme-catalyzed antioxidant system mainly improve the activity of antioxidant enzymes which include peroxidase (POD), superoxide dismutase (SOD), catalase (CAT), and ascorbate peroxidase (APX) (Ezaki et al., 2013), or increase the expression level of antioxidant enzyme genes (Irany et al., 2018). While the non-enzymatic antioxidants are ascorbate (AsA) and glutathione (GSH) (Xu et al., 2012). Ectopic overexpression of wheat *WMnSOD1* and alternative oxidase gene improved Al tolerance in transgenic *Brassica napus* plants and tobacco cells, respectively (Basu et al., 2001; Panda et al., 2013). Transgenic Arabidopsis plants, which overexpressed three glutathione S-transferase genes and two peroxidase genes of tobacco, were endowed with strong aluminum tolerance (Ezaki et al., 2000). Besides, it was also reported some upstream gene, such as *OsPIN2*, and *PEPC* and *PPDK*, which are in positive control of the expression of antioxidant enzyme gene, could also enhance the tolerance of transgenic rice plants to Al toxicity *via* reducing the production of ROS, improving the activity ROS-scavenging enzyme, and weakening lipid peroxidation (Wu et al., 2014; Zhang et al., 2018). Additionally, it was also believed that the synthesis of cysteine-rich proteins can reduce the production of ROS. Fortunately, one of this protein had been identified as an Al tolerance gene that regulated the transcription by STOP1-like protein (ART1) in rice (Xia et al., 2013).

*ZmAT6* (aluminum tolerance 6) gene, which encodes an unknown protein and is upregulated by Al stress, was identified from gene chip data in our previous study (Xu et al., 2017). In this study, we aimed to investigate the role that *ZmAT6* played during Al stress. The Al tolerance-related phenotype of *ZmAT6* was assessed in transgenic maize plants and Arabidopsis, as well as various indices of Al tolerance. The mechanism underlining the involvement of ROS scavenging in the *ZmAT6*-mediated antagonization of Al toxicity was explored.

## Materials and Methods

### Plant Materials and Growth Conditions

The Al-tolerant maize inbred line 178, which has a high value of relative root growth (RRG = 45%) in our previous study (Xu et al., 2017), was used in this study. The seeds of 178 were first sterilized with 75% (w/v) alcohol for 2 min, then with 2% (w/v) NaOCl for 8 min, and finally germinated in quartz sand for 7 d under 28°C, 60% relative humidity and a photoperiod of 16 h/8 h (light/dark) cycle. After germinating, the seedlings were transferred to Hoagland’s solution (Hoagland and Arnon, 1950) and grown for 5 days to the three-leaf stage for further treatment. The nutrient solution was adjusted to pH 5.8 with HCl and renewed every two days. The greenhouse conditions were now set as 14 h/28°C and 10 h/22°C day–night cycle, 70% relative humidity and 300 μmol m^−2^s^−1^ intense luminosity.

### Sequence Characteristic Analysis of *ZmAT6*

Multiple sequence alignment of ZmAT6 (GRMZM5G886177) and its homologs was performed using DNAMAN (LynonBiosoft). Promoter analysis was performed by PlantCARE (http://bioinformatics.psb.ugent.be/webtools/plantcare/html/), and the *cis*-elements are listed in **Table S2**.

Analysis of the Expression of *ZmAT6* by Semi-Quantitative RT-PCR and Real-Time PCR

The tissues and organs 10 days after pollination, including the roots, stems, leaves, ears, tassels, and kernels, were collected from 178; the seedlings were treated with 0.5 mM CaCl_2_ solution (pH 4.2) at 28°C overnight before treatment with Al and then transferred into the same solution containing additively 60 µM AlCl_3_ (pH 4.2) and treated for 0, 6, 12, 24, 48, and 96 h. All the tissues were immediately frozen in liquid nitrogen and stored at −80°C. Semi-quantitative RT-PCR and real-time PCR were carried out as previously described (Lin et al., 2014). Three biological replicates were performed for each experiment. The primers used for *ZmAT6* were listed in **Table S3**.

### Subcellular Location

The subcellular localization of the *ZmAT6* protein was predicted by the WoLF PSORT program (http://wolfpsort.org) (Horton et al., 2007). Moreover, the full length CDS of the*ZmAT6* gene was ligated to pCAMBIA2300 to establish the ZmAT6:GFP vector.

### Overexpression of the *ZmAT6* Gene in Maize and *Arabidopsis*

The full length coding sequence of the *ZmAT6* gene was amplified and ligated to the CPB vector behind the cauliflower mosaic virus 35S (CaMV35S) promoter to construct the p35S:*ZmAT6* vector. The p35S:*ZmAT6* vector was transformed into immature maize callus of 18-599 by an *Agrobacterium-*mediated method (Huang et al., 2018) and into Arabidopsis by the flowering dip method (Clough and Bent, 1998). The transgenic plants of maize and Arabidopsis were confirmed by PCR amplification and harvested individually. The homozygous seeds of T4 generation were used for future experiments.

### Assessment of the Plant Growth, Relative Root Growth, and Al Content

The seedlings of the transgenic maize and wild type (WT) 18-599 were transferred to Hoagland’s solution with or without 200 μM AlCl_3_ (pH 4.2). After two weeks of culture, the fresh weights of the underground part and the upper part of the ground were measured separately.

The root lengths of the transgenic maize plants and wild type were measured and treated as the initial length after pretreatment with 0.5 mM CaCl_2_ for 12 h. The maize plants were exposed to 60 μM AlCl_3_ for 3 days, the final length of the root was recorded, and the RRG was calculated (Sasaki et al., 2004; Sasaki et al., 2006). The root tips (approximately 1 cm) were collected and measured as previously described to determine the Al content (Osawa and Matsumoto, 2001).

### Measurement of Cell Membrane Integrity, Lipid Peroxidation, and Proline Content

After treatment with 60 μM AlCl_3_ for 24 h, the roots of transgenic maize plants and WT were immediately collected and dyed as described by Baker and Mock (Baker and Mock, 1994). Lipid peroxidation was determined by measuring the MDA content using the thiobarbituric acid method (Cakmak and Horst, 1991). Free proline determination was assessed as described by Bates (Bates et al., 1973).

### Determination of ROS Related to Aluminum Stress

After treatment with 60 μM AlCl_3_ for 24 h, the active oxygen content was measured in the seedlings of transgenic maize and WT (Jana and Choudhuri, 1982). The super oxygen free radical (O_2_^·−^) was measured by the hydroxylamine method (Elstner and Heupel, 1976).

### Antioxidant Enzyme Extractions, Activity Assessment Assay, and Gene Expression Detection

After Al treatment, roots were collected immediately, and 0.5 g of the root sample was homogenized with 50 mM sodium phosphate buffer (pH 7.0) containing 3 ml 1 mM ethylenediaminetetraacetic acid (EDTA). Homogenates were then centrifuged at 4°C for 20 min at 15,365 **g**, and the supernatants were used to determine the enzyme activity. The whole extraction procedure was carried out at 4°C.

The activities of SOD, POD, CAT, and APX were determined as previously described (Aebi, 1974; Giannopolitis and Ries, 1977; Nakano and Asada, 1981; Zheng and Huystee, 1992).

The relative expression of antioxidant enzyme genes in WT and one of the maize transgenic line L5 (**Figure S2**) was detected by RT-PCR, and the primers were listed in **Table S3**.

### Statistical Analysis

All experiments were repeated at least three times, and data were represented as the mean ± SD of the replicates. Student’s t tests at *p* < 0.05 and 0.01 were performed to identify significant differences between observation values using the SPSS21 software. The figures were drawn using the Origin 8.0 software (Origin Lab Corporation, Northampton, MA, USA).

## Results

### Cloning and Molecular Characteristics of the ZmAT6 Sequence

**Figure 1:**
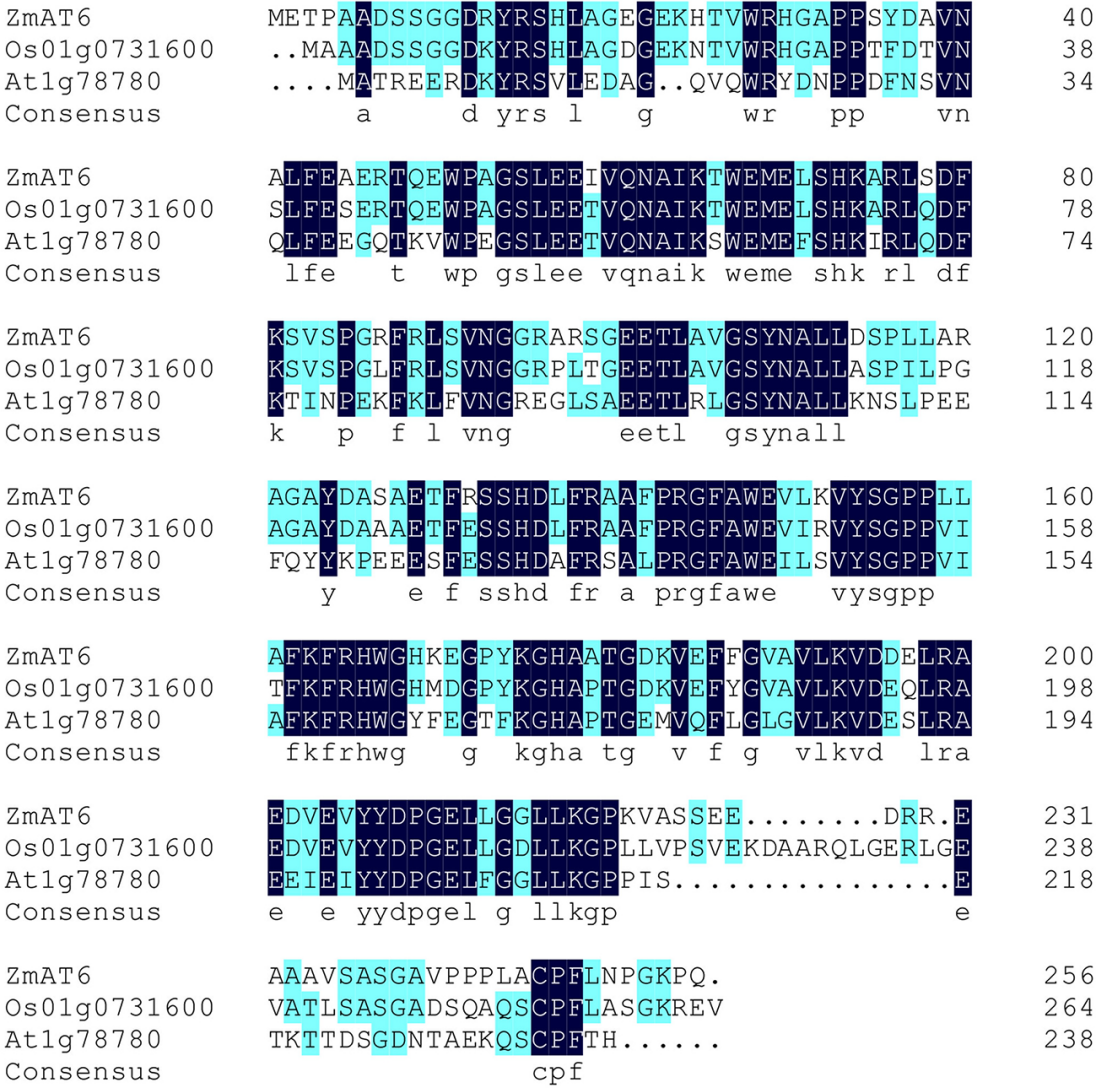
ZmAT6 and its homologs are highly conservative.

Using gene specific primers, the complete CDS sequence of *ZmAT6* was isolated from the aluminum tolerance maize line 178. The 771 bp open reading frame (ORF) of *ZmAT6* has three exons and encodes a 27.79 kDa non-transmembrane protein with a predicted pI of 5.80 (**Figure S1**, **Table S1**). Multiple sequence alignment showed that ZmAT6 shared a higher similarity, up to 75.19%, with the rice homolog (Os01g0731600), higher than its Arabidopsis counterpart (At1g78780) (51.56%) (**Figure 1**). In addition, a promoter analysis of *ZmAT6* revealed that it included but was not limited to the binding sites GGN(T/g/a/C)V(C/A/g)S(C/G) of ART1 (**Table S2**), the major transcriptional factor that regulates the series of Al response genes in rice. However, protein prediction did not reveal any particular structure of ZmAT6.

### Expression Pattern of *ZmAT6* in Different Organs and Under Al Treatment

**Figure 2:**
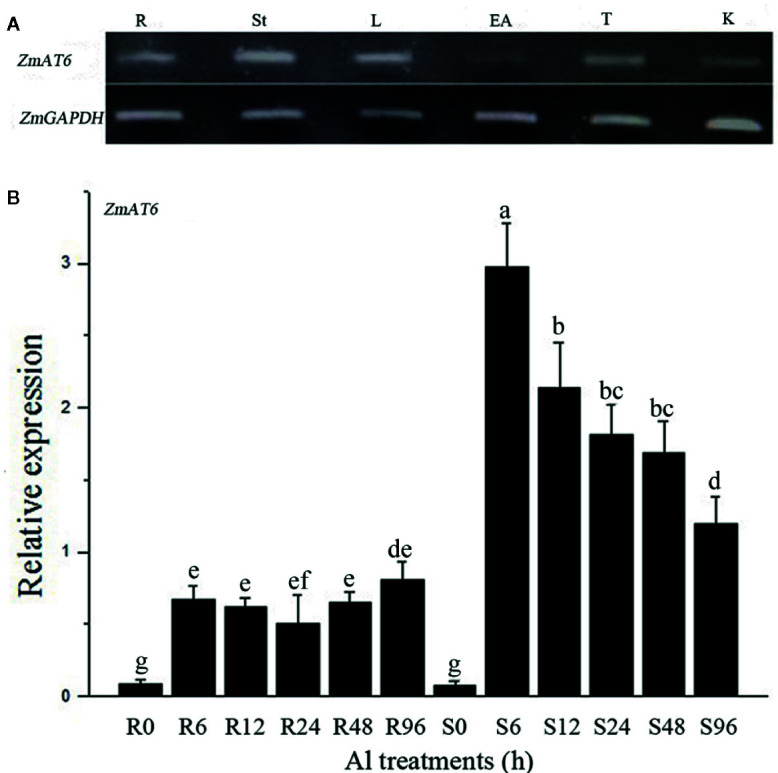
The expression pattern of the *ZmAT6* gene. **(A)** Tissue and organ expression pattern of *ZmAT6* in adult maize plants. The letter above the columns of expression data refer to: R, root; St, stem; L, leaf; EA, ear; T, tassel; and K, kernel. **(B)** Transcription of *ZmAT6* in the roots (R) and shoot (S) was quantified at 0, 6, 12, 24, 48, and 96 h after Al treatment (60 μM AlCl_3_, pH = 4.2). *ZmGAPDH* was used as internal reference gene. The values were presented as mean ± SD (n = 3) and marked with diﬀerent letters to indicate statistic significant diﬀerence at *P* < 0.05 (student’s *t* test).

To better understand the functions of *ZmAT6*, its expression patterns in several organs, including roots, stems, leaves, ears, tassels and kernels, at different growth stages were monitored using semi-quantitative reverse transcription PCR (RT-PCR). *ZmAT6* was moderately expressed in all the organs with the exception of its weak expression in ears (**Figure 2A**). Moreover, to investigate the pattern of expression of *ZmAT6* on Al exposure, the mRNA abundance of *ZmAT6* was monitored further in roots and shoots under a time-course Al stress treatment from 0 to 96 h. As shown in **Figure 2B**, the transcription of *ZmAT6* was dramatically upregulated after Al treatment in the roots or the shoots. The Al-inducted expression level of *ZmAT6* was quite stable in the roots during the whole process. Alternatively, *ZmAT6* in the shoots exhibited a much higher level of expression than in the roots. The mRNA abundance of *ZmAT6* peaked at time point six and then gradually decreased during prolonged Al stress. These results indicated that *ZmAT6* was sensitive to the fluctuating environmental Al^3+^ status in seedlings and was involved in the tolerance to aluminum toxicity in maize.

### Subcellular Localization of ZmAT6

**Figure 3:**
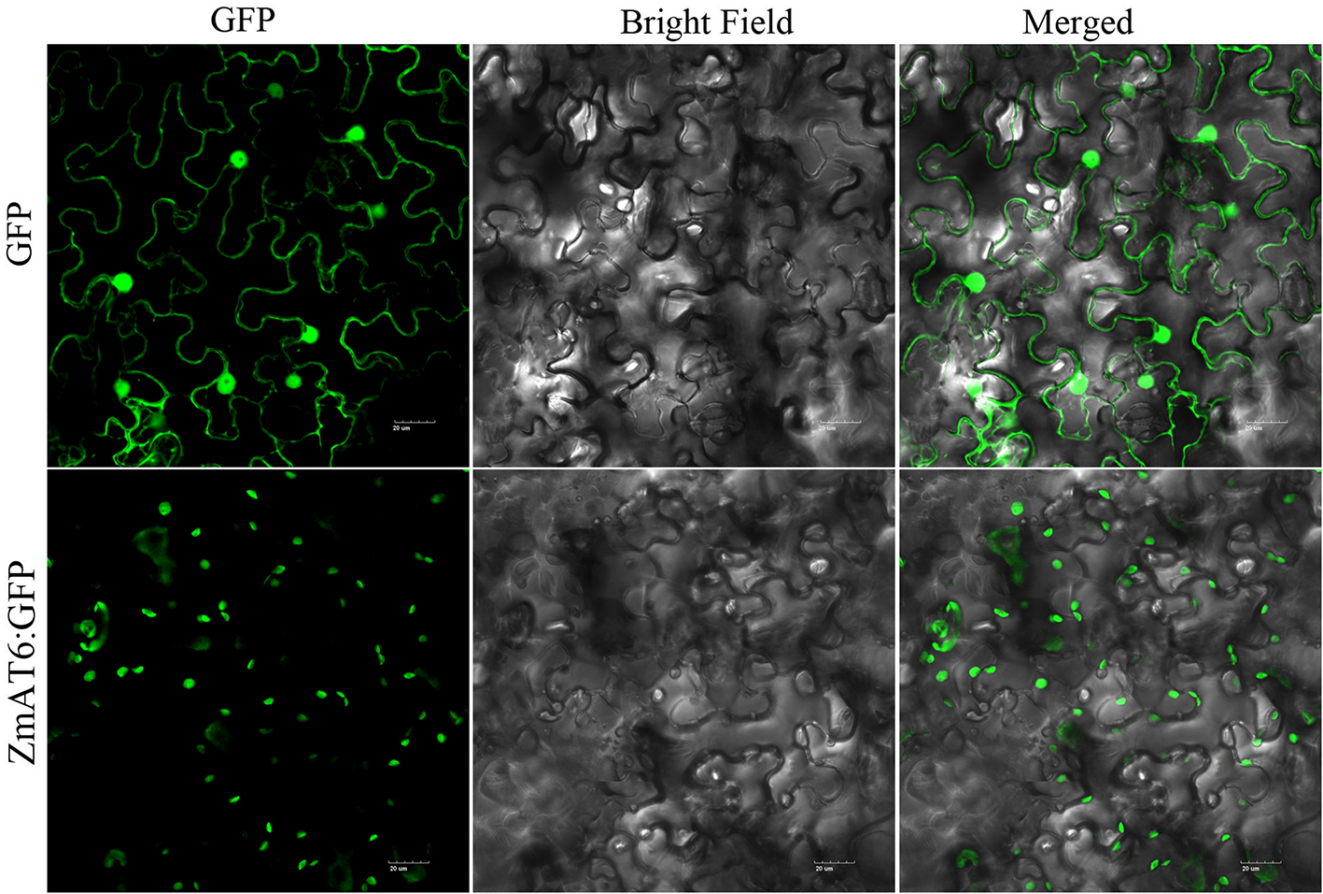
Subcellular localization of ZmAT6.

Using the WoLF PSORT program, ZmAT6 protein was predicted to be localized in the chloroplast or cytoplasm. To detect the subcellular localization of ZmAT6 protein, the coding region of *ZmAT6* was fused with the 3′ end of the *GFP* gene and driven by the *35S* promoter. The *GFP* gene alone under the control of the *35S* promoter served as the control. The subcellular localization of *ZmAT6* was determined in a transient expression system in *Nicotiana benthamiana* leaves. The result indicated that the 35S:ZmAT6:GFP fusion protein appeared to be localized in the chloroplast but not the cytoplasm (**Figure 3**).

### Overexpression of *ZmAT6* in Maize Plants Conferred Improved Al Tolerance

To further investigate the function of *ZmAT6* under Al stress, the entire ORF of *ZmAT6* was inserted into the binary vector CPB (**Figures S2A, B** **)**, and the recombinant expression vector p35S:*ZmAT6* was transformed into maize inbred line 18-599. Finally, 10 independent transgenic plants of T_0_ generation were identified and confirmed by PCR detection (**Figure S2C**). Among them, three homozygous lines (L3, L5, and L10) of T3 generation with higher *ZmAT6* expression (**Figure S2D**) were propagated and selected for analysis.

**Figure 4:**
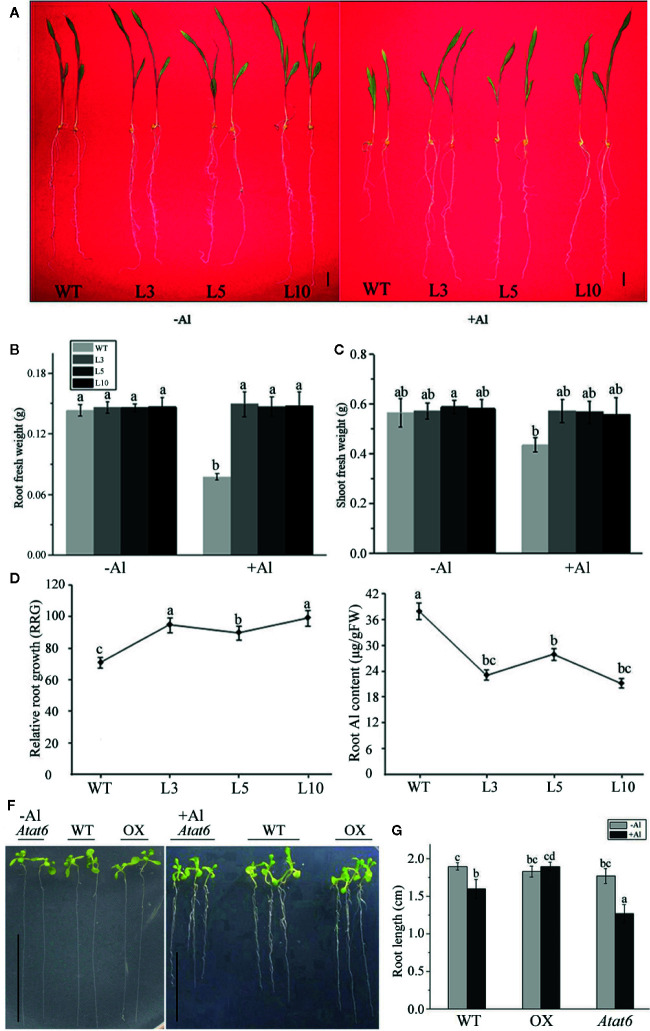
*ZmAT6* overexpression enhanced aluminum tolerance in both transgenic maize and Arabidopsis. **(A)** Wild type (WT) and *OE-ZmAT6* transgenic plants were grown on hydroponic culture with or without Al treatment (60 μM AlCl_3_, pH 4.2). The corresponding **(B)** root fresh weight, **(C)** shoot fresh weight, **(D)** relative root growth (RRG), and **(E)** aluminum (Al) content. Values represent mean ± SD (*n* = 3–5). Scale bar: 2.0 cm. Different letters indicate significant differences (*P* < 0.01) (multiple comparison). **(F)** Wild type Col-0 and mutants *Atat6* and *ZmAT6* overexpressed (OX) transgenic Arabidopsis plants grown with or without aluminum treatment (pH 4.2) and the corresponding **(G)** root length. Scale bar: 1.0 cm. Values represent mean ± SD (*n* ≥ 3). Different letters indicate significant differences (*P* < 0.01) (multiple comparison).

To determine whether the overexpression of *ZmAT6* enhanced the tolerance of the transgenic plants to Al, we assessed the plant growth of the three overexpressed transgenic lines of *ZmAT6* (*OE-ZmAT6*) and the wild type (18-599, WT) under normal and Al stress conditions. At the beginning, the transgenic plants grew normally like the WT in a hydroponic culture (pH 4.2). Nevertheless, when cultivated in the same solution containing 200 μM AlCl_3_, all of the *OE-ZmAT6* plants uniformly showed a high tolerance to Al. The mean value of RRG of *OE-ZmAT6* plants was 90%, much higher than that of the WT (70%) (**Figures 4A, D** **)**. As for the root fresh weight, the mean value of *ZmAT6* transgenic lines was about 87% heavier than that of WT (**Figure 4B**), indicating a less inhibition of *OE-ZmAT6* plants when subjected to Al stress. Moreover, the vigorous leaf growth of the *OE-ZmAT6* transgenic plants could also be verified by the significant increase in the shoot fresh weights (**Figures 4A, C** **)**. Concerning the Al content in root tips, the mean value of 24.63 μg/g fresh weight (FW) was lower in the *OE-ZmAT6* plants than the 37.23μg/g FW in the WT (**Figure 4E**).

In addition, evidence from its Arabidopsis homolog *AtAT6* reinforced the fact that *AtAT6* could enhance or decrease aluminum tolerance *via* overexpression or mutation (**Figure S3**, **Figures 4F, G** **).** In comparison with the WT Col-0, the root length of the *Atat6* mutant (SALK_082224) was much shorter, while those of the *AtAT6*-overexpressed (OX) Arabidopsis plants were much longer on Al exposure (**Figure 4G**).

**Figure 5:**
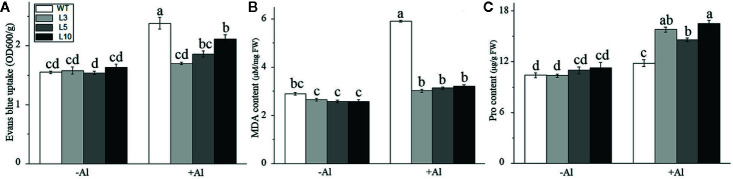
Determination of physiological indexes related to aluminum tolerance. **(A)** Evans blue, **(B)** Malondialdehyde (MDA) and **(C)** Proline (Pro) in the wild type (WT) and three *OE-ZmAT6* transgenic maize lines (L3, l5, and L10). Values represent mean ± SD (*n* = 10). Different letters indicate significant differences (*P* < 0.01) (multiple comparison).

Furthermore, physiological indices related to Al-stress tolerance, including Evans blue staining, malondialdehyde (MDA) content, and proline (Pro) content, were also measured in three *OE-ZmAT6* transgenic lines and WT maize plants on Al exposure. As shown in **Figure 5** and **Figure S4**, the results indicated that the mean values of the Evans blue uptake (1.80 OD 600/g) and MDA content (3.14 μM/μg FW) in the *OE-ZmAT6* lines were significantly lower than those of the WT under Al treatment. In contrast, the Pro content (15.80 μM/g FW) was clearly higher than that in the WT (11.77 μM/g FW) when the plants suffered from Al toxicity (**Figure 5C**). These results verified the fact that overexpression of *ZmAT6* conferred tolerance to Al toxicity in *OE-ZmAT6* plants and reduced the oxidative damage of maize under Al stress.

### *ZmAT6* Is Related to the Scavenging of ROS

**Figure 6:**
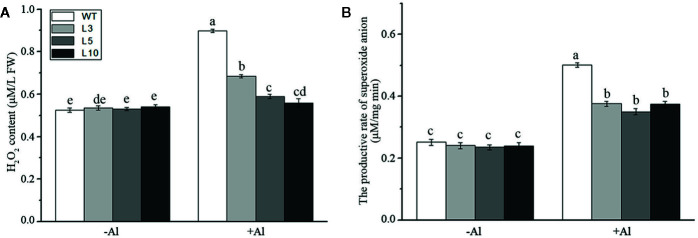
Determination of ROS under aluminum stress. **(A)** Hydrogen peroxide content and **(B)** the productive rate of superoxide anion in wild type (WT) and three *OE-ZmAT6* transgenic maize lines (L3, L5, and L10). Values represent mean ± SD (*n* = 3). Different letters indicate significant differences (*P* < 0.01) (multiple comparison).

To investigate whether *ZmAT6* is related to the scavenging of ROS, we measured the content of hydrogen peroxide (H_2_O_2_) and the rate of production of the superoxide anion in the three *OE-ZmAT6* lines and the WT. Under normal conditions, no distinct difference could be detected between the *OE-ZmAT6* lines and WT regarding these two indices. Nevertheless, both H_2_O_2_ and the superoxide anion had increased in all the plants tested during Al stress, and the amplitude was even higher in the WT than in *OE-ZmAT6* plants (**Figures 6A, B**).The increase of H_2_O_2_ content and productive rate of superoxide anion in WT against *OE-ZmAT6* lines under Al stress were 0.38:0.09 (μM/L FW) and 0.24:0.11 (μM/mg) min, respectively. These results indicated that *ZmAT6* played an important role in the scavenging of the excessive ROS caused by Al stress, which endowed transgenic maize plants with the capacity to tolerate aluminum toxicity by ROS cleaning.

### *ZmAT6* Improved the Activity of Antioxidant Enzymes and the Expression Level of Antioxidant Genes in Transgenic Maize

**Figure 7:**
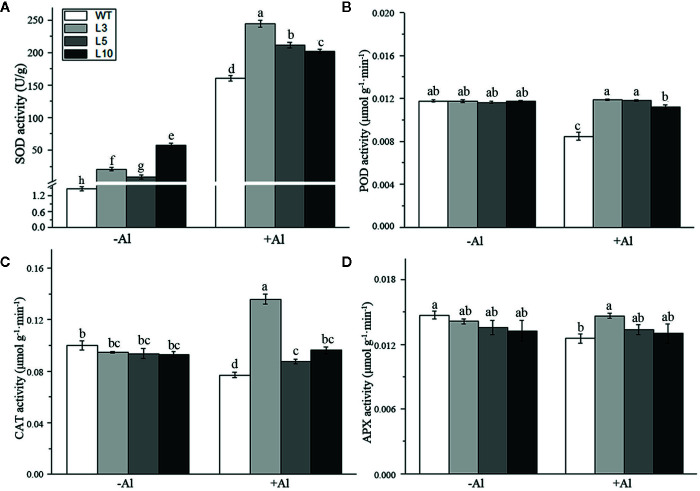
Determination of the activity of several antioxidant enzymes. The activities of antioxidant-related enzymes **(A)** SOD, **(B)** POD, **(C)** CAT, and **(D)** APX were detected. Values represent mean ± SD (*n* = 4). Different letters indicate significant differences (*P* < 0.05) (multiple comparison).

To investigate the factors affecting the *ZmAT6*-mediated scavenging of ROS, we examined the activity of several enzymes usually involved in the antioxidant system. It was notable that the SOD activity of three *OE-ZmAT6* lines was significantly higher than that of the WT despite Al treatment. After treatment with 60 μM AlCl_3_ (pH 4.2), the mean value of SOD activity of the *OE-ZmAT6* plants was dramatically increased, up to 244.4 U/g (**Figure 7A**). The activities of POD, CAT, and APX remained almost the same in the WT plants as in the *OE-ZmAT6* plants but decreased separately to 28.07, 22.92, and 14.77%, respectively, when subjected to Al stress (**Figures 7B–D**).

**Figure 8:**
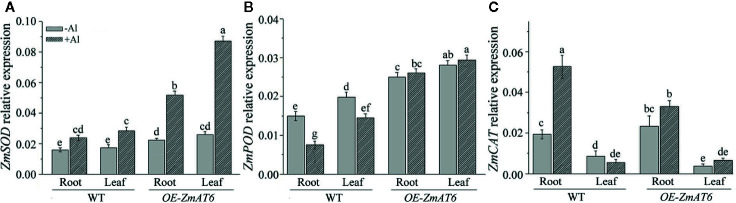
The relative expression of antioxidant enzyme genes. The relative expression levels of **(A)** *ZmSOD*, **(B)** *ZmPOD*, and **(C)** *ZmCAT* were determined with or without Al treatment in WT and *OE-ZmAT6* transgenic maize (L5). *ZmGAPDH* was used as internal reference gene. Values represent mean ± SD (n = 3). Different letters indicate significant differences (*P* < 0.05) (multiple comparison).

In addition, we also monitored the expression patterns of three antioxidant-enzyme genes on Al exposure. As shown in **Figures 8A, B**, the expression of *ZmSOD* and *ZmPOD* could be upregulated by Al stimulus in all tested plants with the exception of *ZmPOD* in WT, consistent with the enhanced activities of antioxidant enzymes (**Figure 7**). As for *ZmSOD* and *ZmPOD*, both of them exhibited a much higher level of expression either in the root or leaf of *OE-ZmAT6* plant than in WT. In particular, the expression of *ZmCAT* in the root of WT was higher than in *OE-ZmAT6* plant (**Figure 8C**). These results suggested that both the regulation on gene expression and activity of antioxidant enzymes contributed to the enhanced Al tolerance of *OE-ZmAT6* plant in maize.

## Discussion

### *ZmAT6* Is Involved in Al-Stress Related Responses

Some plants evolved many mechanisms to tolerate Al toxicity. *ZmAT6* was first identified as an Al-induced gene with an unpredicted function from our previous microarray data (Xu et al., 2017). The unique indication was that its rice or tholog was regulated by OsART1, a master transcriptional factor that controls more than 30 Al response genes by binding to the GGTCC (GGN(T/g/a/C)V(C/A/g)S(C/G)) site of its promoter (Yamaji et al., 2009; Tsutsui et al., 2011). Similarly, we also found a GGNVS site in the promoter of *ZmAT6* (**Table S2**). In addition, *ZmAT6* expresses in many tissues and organs, and it could be rapidly induced by Al stress in both the roots and shoots of maize (**Figure 2**). These results suggested that *ZmAT6* may be a downstream gene in maize directly targeted by a transcription factor such as rice OsART1 when the plants are under Al stress. An exploration of the OsART1 equivalent in maize by strategies such as homolog query and gel shift assay could confirm this hypothesis.

### Overexpression of *ZmAT6* Improved the Al Tolerance of Transgenic Maize Plants

The inhibition of the root length was an initial detection of Al toxicity in plants (Matsumoto and Motoda, 2012). Plants with strong Al tolerance usually exhibited high relative root growth (Ma et al., 2018; Badia et al., 2020). In this study, the RRG of *OE-ZmAT6* transgenic maize was more than 20% higher than those in the WT. Meanwhile, the mean value of the total fresh weight and the fresh weight of roots were more than 30% and even over 50% weightier than that of WT after exposure to Al, respectively (**Figures 4A–D**). Attributed to the ectopic overexpression of *ZmAT6*, the clear improvement of the Al tolerance was exhibited not only in transgenic maize plants but also in transgenic wild type Arabidopsis and *at6* mutant (**Figures 4F, G** **)**. Moreover, the content of aluminum in the root of *OE-ZmAT6* plant is rather lower than that in the WT (**Figure 4E**), underlying an antagonism to Al toxicity.

In addition, a semi reduction of the superoxide radical ion caused by aluminum toxicity may lead to oxidative stress and serious cell damage. The enzyme-catalyzed antioxidant system and the non-enzymatic antioxidant system of plants could scavenge excessive ROS and reduce the cell damage caused by aluminum toxicity (Achary et al., 2008; Irany et al., 2018). The intensity of Evans blue staining and the content of MDA in the cells of target tissue could reflect the lipid peroxidation of the cell membrane. In this study, the significantly lower Evans blue uptake (**Figure 5A**, **Figure S4**) and MDA content (**Figure 5B**) of the *OE-ZmAT6* plants suggested that aluminum stress caused less damage to the cell membrane. This was consistent with the results of Sun (Sun et al., 2017) and Yu (Yu et al., 2018), who reported that the MDA content in the root tips of wheat increased significantly under aluminum treatment, particularly in sensitive genotype plants, and the higher Evans Blue uptake in the root apexes was due to the Al-induced oxidative stress. Moreover, proline, a type of antioxidant that favors ROS removal (Rodriguez and Redman, 2005), had a significantly higher content in tolerant plants than in sensitive ones (Ashraf and Foolad, 2007). The obviously increased content of proline in the *OE*-*ZmAT6* plants under Al stress in this study was highly consistent with the previous study of Giannakoula (Giannakoula et al., 2008), who found that the proline content in the Al-tolerant genotype maize increased with the concentration of Al.

### Scavenging of ROS Favors *OE-ZmAT6* Maize Plants to Antagonize Al-Stress

Previous studies have documented that the excessive accumulation of ROS in plants under aluminum stress is the primary reason for oxidative stress and the decisive factor that inhibited root elongation (Tamás et al., 2006; Giannakoula et al., 2010). Giannakoula (Giannakoula et al., 2010) and Sun (Sun et al., 2017) had found that O_2_^·−^ and H_2_O_2_ could predominantly accumulate in the roots of Al-sensitive maize and wheat, respectively, after exposure to Al. Compared with the wild type, overexpression of *AtPrx64* in transgenic tobacco also showed less root growth inhibition, lower H_2_O_2_ content, and less MDA accumulation following Al exposure (Wu et al., 2017). In our study, the content of H_2_O_2_ and the productive rate of the superoxide anion in the ROS of *OE-ZmAT6* maize plants were significantly lower than those in the WT under Al stress (**Figure 6**), indicating the involvement of AT6 in ROS scavenging. Indeed, the activities of the antioxidant enzymes POD, CAT, and APX of the *OE-ZmAT6* plants had no obvious change before and after Al treatment even though there was a significant difference between the transgenic lines and WT (**Figures 7B–D**). The only exception was SOD, which had significantly higher activities in the *OE-ZmAT6* plants than that in the WT before or after Al stress (**Figure 7A**). Consistently, the expression level of *ZmSOD* was also predominately upregulated by Al stimulus (**Figure 8A**), suggesting that SOD played a crucial role in effectively removing excess ROS from *OE-ZmAT6* maize cells and maintaining the balance of ROS.

Coincidentally, a number of studies also found that the enzyme-catalyzed antioxidant system is involved in Al stress antagonism even though the major scavenger enzyme was not the same. Darkó et al. (Darkó et al., 2004) found that the activity of the antioxidant enzymes in the wheat root tips changed noticeably under Al stress, and the activity of APX in Al-sensitive genotypes was significantly higher, while those of SOD and CAT were lower than those of the Al-tolerant genotype. However, the activities of SOD and POD were significantly improved in Al-sensitive maize after Al treatment, while the activities of SOD, POD and CAT exhibited no distinction in the Al-tolerant genotypes (Boscolo et al., 2003). Therefore, the ROS scavenging responses of antioxidant enzymes under Al stress conditions varied depending on the species and genotypes. Alternatively, the overexpression of genes encoding ROS-scavenging enzymes in several plant species had documented that their activities enhanced the tolerance of Al (Panda et al., 2013; Sun et al., 2017; Irany et al., 2018). In this study, *ZmAT6* enhanced the aluminum tolerance of maize by increasing the expression level of *ZmSOD* gene and improving the activity of the antioxidant enzymes SOD in the antioxidant enzymatic system.

In conclusion, we demonstrated that the chloroplast-located gene *ZmAT6* could be quickly induced by Al stress and could enhance the tolerance to Al toxicity when overexpressed in transgenic maize and Arabidopsis. *ZmAT6* could antagonize the Al toxicity *via* at least two ROS removal approaches: increasing the activity of antioxidant enzymes SOD and the content of antioxidant proline.

## Data Availability Statement

The raw data supporting the conclusions of this article will be made available by the authors, without undue reservation.

## Author Contributions

HD and YH carried out the experiments, analyzed the data, and drafted the manuscript. JD, HL, XH, MQ, YLi, and WY contributed to sample collection and data analysis. WH, MC, SG, and YLu contributed with consultation. SZ designed the experiment and revised the manuscript. All authors contributed to the article and approved the submitted version.

## Funding

This work was supported by the National Natural Science Foundation of China [No. 30800687, 31071434], and the Major Project of Education Department in Sichuan [No. 15ZA0022].

## Conflict of Interest

The authors declare that the research was conducted in the absence of any commercial or financial relationships that could be construed as a potential conflict of interest.

## References

[B1] Achary V. M. Jena S. Panda K. K. Panda B. B. (2008). Aluminium induced oxidative stress and DNA damage in root cells of *Allium cepa* L. Ecotoxicol. Environ. Saf. 70, 300–310. 10.1016/j.ecoenv.2007.10.022 18068230

[B2] Aebi H. (1974). “Catalase, method of enzymatic analysis,” in Method of enzymatic analysis 3. Ed. Bergmeyer H. U. (New York: Academic), 673–684.

[B3] Ahmad P. Jaleel C. A. Salem M. A. Nabi G. Sharma S. (2010). Roles of enzymatic and nonenzymatic antioxidants in plants during abiotic stress. Crit. Rev. Biotechnol. 30, 161–175. 10.3109/07388550903524243 20214435

[B4] Arenhart R. A. Schunemann M. Bucker Neto L. Margis R. Wang Z. Y. Margis-Pinheiro M. (2016). Rice ASR1 and ASR5 are complementary transcription factors regulating aluminium responsive genes. Plant Cell Environ. 39, 645–651. 10.1111/pce.12655 26476017 PMC7256019

[B5] Ashraf M. Foolad M. R. (2007). Roles of glycine betaine and proline in improving plant abiotic stress resistance. Environ. Exp. Bot. 59, 206–216. 10.1016/j.envexpbot.2005.12.006

[B6] Badia M. B. Maurino V. G. Pavlovic T. Arias C. L. Pagani M. A. Andreo C. S. (2020). Loss of function of Arabidopsis NADP-malic enzyme 1 results in enhanced tolerance to aluminum stress. Plant J. 101, 653–665. 10.1111/tpj.14571 31626366

[B7] Baker C. J. Mock N. M. (1994). An improved methods for monitoring cell death in cell suspension and leaf disc assays using evans blue. Plant Cell Tiss. Org. Cult. 39, 7–12. 10.1007/BF00037585

[B8] Basu U. Good A. G. Taylor G. J. (2001). Transgenic Brassica napus plants overexpressing aluminum-induced mitochondrial manganese superoxide dismutase cDNA are resistant to aluminum. Plant Cell Environ. 24, 1278–1269. 10.1046/j.0016-8025.2001.00783.x

[B9] Bates L. S. Waldren R. P. Teare I. D. (1973). Rapid determination of free proline for water-stress studies. Plant Soil 39, 205–207. 10.1007/BF00018060

[B10] Boscolo P. R. S. Menossi M. Jorge R. A. (2003). Aluminum-induced oxidative stress in maize. Phytochemistry 62, 181–189. 10.1016/S0031-9422(02)00491-0 12482454

[B11] Cakmak I. Horst W. J. (1991). Effect of aluminium on lipid peroxidation, superoxide dismutase, catalase, and peroxidase activities in root tips of soybean (*Glycine max*). Physiol. Plant. 83, 463–468. 10.1111/j.1399-3054.1991.tb00121.x

[B12] Chowra U. Yanase E. Koyama H. Panda S. K. (2017). Aluminium-induced excessive ROS causes cellular damage and metabolic shifts in black gram *Vigna mungo* (L.) Hepper. Protoplasma 254, 293–302. 10.1007/s00709-016-0943-5 26769708

[B13] Clough S. J. Bent A. F. (1998). Floral dip: a simplifed method for Agrobacterium -mediated transformation of *Arabidopsis thaliana* . Plant J. Cell Mol. Biol. 16, 735. 10.1046/j.1365-313x.1998.00343.x 10069079

[B14] Darkó É Ambrus H. Stefanovits-Bányai É Fodor J. Bakos F. Barnabás B. (2004). Aluminium toxicity, Al tolerance and oxidative stress in an Al-sensitive wheat genotype and in Al-tolerant lines developed by in vitro microspore selection. Plant Sci. 166, 583–591. 10.1016/j.plantsci.2003.10.023

[B15] Daspute A. A. Sadhukhan A. Tokizawa M. Kobayashi Y. Panda S. K. Koyama H. (2017). Transcriptional regulation of aluminum-tolerance genes in higher plants: clarifying the underlying molecular mechanisms. Front. Plant Sci. 8, 1358. 10.3389/fpls.2017.01358 28848571 PMC5550694

[B16] Ding Z. J. Yan J. Y. Xu X. Y. Li G. X. Zheng S. J. (2013). WRKY46 functions as a transcriptional repressor of ALMT1, regulating aluminum-induced malate secretion in Arabidopsis. Plant J. 76, 825–835. 10.1111/tpj.12337 24118304

[B17] Elstner E. F. Heupel A. (1976). Inhibition of nitrite formation from hydroxylammoniumchloride: a simple assay for superoxide dismutase. Anal. Biochem. 70, 616–620. 10.1016/0003-2697(76)90488-7 817618

[B18] Exley C. (2004). The pro-oxidant activity of aluminum. Free Radic. Biol. Med. 36, 380–387. 10.1016/j.freeradbiomed.2003.11.017 15036357

[B19] Ezaki B. Gardner R. C. Ezaki Y. Matsumoto H. (2000). Expression of aluminum-induced genes in transgenic Arabidopsis plants can ameliorate aluminum stress and/or oxidative stress. Plant Physiol. 122, 657–665. 10.1104/pp.122.3.657 10712528 PMC58900

[B20] Ezaki B. Jayaram K. Higashi A. Takahashi K. (2013). A combination of five mechanisms confers a high tolerance for aluminum to a wild species of Poaceae, *Andropogon virginicus* L. Environ. Exp. Bot. 93, 35–44. 10.1016/j.envexpbot.2013.05.002

[B21] Furukawa J. Yamaji N. Wang H. Mitani N. Murata Y. Sato K. (2007). An aluminum-activated citrate transporter in barley. Plant Cell Physiol. 48, 1081–1091. 10.1093/pcp/pcm091 17634181

[B22] Giannakoula A. Moustakas M. Mylona P. Papadakis I. Yupsanis T. (2008). Aluminum tolerance in maize is correlated with increased levels of mineral nutrients, carbohydrates and proline, and decreased levels of lipid peroxidation and Al accumulation. J. Plant Physiol. 165, 385–396. 10.1016/j.jplph.2007.01.014 17646031

[B23] Giannakoula A. Moustakas M. Syros T. Yupsanis T. (2010). Aluminum stress induces up-regulation of an efficient antioxidant system in the Al-tolerant maize line but not in the Al-sensitive line. Environ. Exp. Bot. 67, 487–494. 10.1016/j.envexpbot.2009.07.010

[B24] Giannopolitis C. N. Ries S. K. (1977). Superoxide dismutases I. Occurrence in higher plants. Plant Physiol. 59, 309–314. 10.1104/pp.59.2.309 16659839 PMC542387

[B25] Hoagland D. R. Arnon D. II (1950). The water-culture method for growing plants without soil. Univ. Galif. Agric. Exp. Sta. Circ. 347, 357–359. 10.1016/S0140-6736(00)73482-9

[B26] Hoekenga O. A. Maron L. G. Piñeros M. A. Cancado G. M. Shaff J. Kobayashi Y. (2006). *AtALMT1*, which encodes a malate transporter, is identified as one of several genes critical for aluminum tolerance in Arabidopsis. PNAS 103, 9738–9743. 10.1073/pnas.0602868103 16740662 PMC1480476

[B27] Horton P. Park K. J. Obayashi T. Fujita N. Harada H. Adams-Collier C. J. (2007). WoLF PSORT: protein localization predictor. Nucleic Acids Res. 35, 585–587. 10.1093/nar/gkm259 PMC1933216 17517783

[B28] Huang C. F. Yamaji N. Chen Z. Ma J. F. (2012). A tonoplast-localized half-size ABC transporter is required for internal detoxification of aluminum in rice. Plant J. 69, 857–867. 10.1111/j.1365-313X.2011.04837.x 22035218

[B29] Huang S. Gao J. You J. Liang Y. Guan K. Yan S. (2018). Identification of STOP1-like proteins associated with aluminum tolerance in sweet sorghum (*Sorghum bicolor* L.). Front. Plant Sci. 9, 258. 10.3389/fpls.2018.00258 29541086 PMC5835670

[B30] Irany R. P. Anny C. D. L. Tatiane L. P. Raquel A. A. Maria D. C. P. B. (2018). Gene expression and antioxidant enzymatic activity in passion fruit exposed to aluminum. Afr. J. Agric. Res. 13, 115–120. 10.5897/ajar2017.12834

[B31] Iuchi S. Koyama H. Iuchi A. Kobayashi Y. Kitabayashi S. Ikka T. (2007). Zinc finger protein STOP1 is critical for proton tolerance in Arabidopsis and coregulates a key gene in aluminum tolerance. PNAS 104, 9900–9905. 10.1073/pnas.0700117104 17535918 PMC1887543

[B32] Jana S. Choudhuri M. A. (1982). Glycolate metabolism of three submersed aquatic angiosperms during ageing. Aquat Bot. 12, 345–354. 10.1016/0304-3770(82)90026-2

[B33] Keith D. Richards E. J. S. Yogesh K. S. Keith R. D. Richard C. G. (1998). Aluminum induces oxidative stress genes in *Arabdopsis thaliana* . Plant Physiol. 116, 409–418. 10.1104/pp.116.1.409 9449849 PMC35183

[B34] Kinraide T. B. (1990). Assessing the rhizotoxicity of the aluminate ion, Al(OH)^4–^ . Plant Physiol. 93, 1620–1625. 10.1104/pp.93.4.1620 16667665 PMC1062720

[B35] Kochian L. V. Hoekenga O. A. Pineros M. A. (2004). How do crop plants tolerate acid soils? Mechanisms of aluminum tolerance and phosphorous efficiency. Annu. Rev. Plant Biol. 55, 459–493. 10.1146/annurev.arplant.55.031903.141655 15377228

[B36] Kochian L. V. Piñeros M. A. Hoekenga O. A. (2005). The physiology, genetics and molecular biology of plant aluminum resistance and toxicity. Plant Soil 274, 175–195. 10.1007/s11104-004-1158-7

[B37] Kochian L. V. Pineros M. A. Liu J. Magalhaes J. V. (2015). Plant adaptation to acid soils: The molecular basis for crop aluminum resistance. Annu. Rev. Plant Biol. 66, 571–598. 10.1146/annurev-arplant-043014-114822 25621514

[B38] Kochian L. V. (1995). Cellular mechanisms of aluminum toxicity and resistance in plants. Annu. Rev. Plant Biol. 46, 237–260. 10.1146/annurev.pp.46.060195.001321

[B39] Li J. Y. Liu J. Dong D. Jia X. McCouch S. R. Kochian L. V. (2014). Natural variation underlies alterations in Nramp aluminum transporter (NRAT1) expression and function that play a key role in rice aluminum tolerance. Proc. Natl. Acad. Sci. U. S. A. 111, 6503–6508. 10.1073/pnas.1318975111 24728832 PMC4035919

[B40] Li G. Z. Wang Z. Q. Yokosho K. Ding B. Fan W. Gong Q. Q. (2018). Transcription factor WRKY22 promotes aluminum tolerance via activation of OsFRDL4 expression and enhancement of citrate secretion in rice (Oryza sativa). New Phytol. 219, 149–162. 10.1111/nph.15143 29658118

[B41] Lin Y. A. Zhang C. L. Lan H. Gao S. B. Liu H. L. Liu J. (2014). Validation of potential reference genes for qPCR in maize across abiotic stresses, hormone treatments, and tissue types. PloS One 9, e95445. 10.1371/journal.pone.0095445 24810581 PMC4014480

[B42] Liu M. Y. Chen W. W. Xu J. M. Fan W. Yang J. L. Zheng S. J. (2013). The role of *VuMATE1* expression in aluminium-inducible citrate secretion in rice bean (*Vigna umbellata*) roots. J. Exp. Bot. 64, 1795–1804. 10.1093/jxb/ert039 23408830 PMC3638816

[B43] Lou H. Q. Fan W. Jin J. F. Xu J. M. Chen W. W. Yang J. L. (2019). A NAC-type transcription factor confers aluminium resistance by regulating cell wall-associated receptor kinase 1 and cell wall pectin. Plant Cell Environ. 43, 463–478. 10.1111/pce.13676 31713247

[B44] Lu M. Wang Z. Fu S. Yang G. Shi M. Lu Y. (2017). Functional characterization of the *SbNrat1* gene in sorghum. Plant Sci. 262, 18–23. 10.1016/j.plantsci.2017.05.010 28716414

[B45] Ma J. F. Furukawa J. (2003). Recent progress in the research of external Al detoxification in higher plants: a minireview. J. Inorg. Biochem. 97, 46–51. 10.1016/s0162-0134(03)00245-9 14507459

[B46] Ma J. F. Hiradate S. Nomoto K. Iwashlta T. Matsumoto H. (1997). Internal detoxification mechanism of Al in hydrangea (identification of Al form in the leaves). Plant Physiol. 113, 1033–1039. 10.1104/pp.113.4.1033 12223659 PMC158226

[B47] Ma Q. Yi R. Li L. Liang Z. Zeng T. Zhang Y. (2018). *GsMATE* encoding a multidrug and toxic compound extrusion transporter enhances aluminum tolerance in. Arabidopsis Thaliana BMC Plant Biol. 18, 212. 10.1186/s12870-018-1397-z 30268093 PMC6162897

[B48] Ma J. F. (2000). Role of organic acids in detoxification of aluminum in higher plants. Plant Cell Physiol. 41, 383–390. 10.1093/pcp/41.4.383 10845450

[B49] Matsumoto H. Motoda H. (2012). Aluminum toxicity recovery processes in root apices. Possible association with oxidative stress. Plant Sci. 185–186, 1–8. 10.1016/j.plantsci.2011.07.019 22325861

[B50] Nakano Y. Asada K. (1981). Hydrogen peroxide is pcavenged by ascorbate-specific peroxidase in spinach chloroplasts. Plant Cell Physiol. 22, 867–880. 10.1093/oxfordjournals.pcp.a076232

[B51] Osawa H. Matsumoto H. (2001). Possible involvement of protein phosphorylation in aluminum-responsive malate efflux from wheat root apex. Plant Physiol. 126, 411–420. 10.1104/pp.126.1.411 11351103 PMC102314

[B52] Panda S. K. Sahoo L. Katsuhara M. Matsumoto H. (2013). Overexpression of alternative oxidase gene confers aluminum tolerance by altering the respiratory capacity and the response to oxidative stress in tobacco cells. Mol. Biotechnol. 54, 551–563. 10.1007/s12033-012-9595-7 22965419

[B53] Rodriguez R. Redman R. (2005). Balancing the generation and elimination of reactive oxygen species. PNAS 102, 3175–3176. 10.1073/pnas.0500367102 15728396 PMC552941

[B54] Sagi M. Fluhr R. (2001). Superoxide production by plant homologues of the gp91(phox) NADPH oxidase. Modulation of activity by calcium and by tobacco mosaic virus infection. Plant Physiol. 126, 1281–1290. 10.1104/pp.126.3.1281 11457979 PMC116485

[B55] Sasaki T. Yamamoto Y. Ezaki B. Katsuhara M. Ahn S. J. Ryan P. R. (2004). A wheat gene encoding an aluminum-activated malate transporter. Plant J. 37, 645–653. 10.1111/j.1365-313X.2003.01991.x 14871306

[B56] Sasaki T. Ryan P. R. Delhaize E. Hebb D. M. Ogihara Y. Kawaura K. (2006). Sequence upstream of the wheat (Triticum aestivum L.) ALMT1 gene and its relationship to aluminum resistance. Plant Cell Physiol. 47, 1343–1354. 10.1093/pcp/pcl002 16928694

[B57] Šimonovičová M. Huttová J. Mistrík I. Široká B. Tamás L. (2004). Root growth inhibition by aluminum is probably caused by cell death due to peroxidase-mediated hydrogen peroxide production. Protoplasma 224, 91–98. 10.1007/s00709-004-0054-6 15726813

[B58] Sun C. Liu L. Zhou W. Lu L. Jin C. Lin X. (2017). Aluminum Induces Distinct Changes in the Metabolism of Reactive Oxygen and Nitrogen Species in the Roots of Two Wheat Genotypes with Different Aluminum Resistance. J. Age Food Chem. 65, 9419–9427. 10.1021/acs.jafc.7b03386 29016127

[B59] Tamás L. Huttova J. Mistrik I. Simonovicova M. Siroka B. (2006). Aluminium-induced drought and oxidative stress in barley roots. J. Plant Physiol. 163, 781–784. 10.1016/j.jplph.2005.08.012 16616589

[B60] Taylor G. J. McDonald-Stephens J. L. Hunter D. B. Bertsch P. M. Elmore D. Rengel Z. (2000). Direct Measurement of Aluminum Uptake and Distribution in Single Cells of. Chara Corallina Plant Physiol. 123, 987–996. 10.1104/pp.123.3.987 10889247 PMC59061

[B61] Tsutsui T. Yamaji N. Ma J. F. (2011). Identification of a cis-acting element of ART1, a C2H2-type zinc-finger transcription factor for aluminum tolerance in rice. Plant Physiol. 156, 925–931. 10.1104/pp.111.175802 21502187 PMC3177286

[B62] von Uexküll H. R. Mutert E. (1995). Global extent, development and economic-impact of acid soils. Plant Soil 171, 1–15. 10.1007/bf00009558

[B63] Wang Y. Li R. Li D. Jia X. Zhou D. Li J. (2017). NIP1;2 is a plasma membrane-localized transporter mediating aluminum uptake, translocation, and tolerance in Arabidopsis. Proc. Natl. Acad. Sci. U. S. A. 114, 5047–5052. 10.1073/pnas.1618557114 28439024 PMC5441725

[B64] Wu D. Shen H. Yokawa K. Baluska F. (2014). Alleviation of aluminium-induced cell rigidity by overexpression of OsPIN2 in rice roots. J. Exp. Bot. 65, 5305–5315. 10.1093/jxb/eru292 25053643 PMC4157713

[B65] Wu Y. S. Yang Z. L. How J. Y. Xu H. N. Chen L. M. Li K. Z. (2017). Overexpression of a peroxidase gene (AtPrx64) of *Arabidopsis thaliana in* tobacco improves plant’s tolerance to aluminum stress. Plant Mol. Biol. 95, 157–168. 10.1007/s11103-017-0644-2 28815457

[B66] Xia J. Yamaji N. Ma J. F. (2013). A plasma membrane-localized small peptide is involved in rice aluminum tolerance. Plant J. 76, 345–355. 10.1111/tpj.12296 23888867

[B67] Xu F. J. Li G. Jin C. W. Liu W. J. Zhang S. S. Zhang Y. S. (2012). Aluminum-induced changes in reactive oxygen species accumulation, lipid peroxidation and antioxidant capacity in wheat root tips. Biol. Plant 51, 89–96. 10.1007/s10535-012-0021-6

[B68] Xu L. M. Liu W. Cui B. M. Wang N. Ding J. Z. Liu C. (2017). Aluminium Tolerance Assessment of 141 Maize Germplasms in a Solution Culture. Universal J. Agric. Res. 5, 1–9. 10.13189/ujar.2017.050101

[B69] Yamaji N. Huang C. F. Nagao S. Yano M. Sato Y. Nagamura Y. (2009). A zinc finger transcription factor ART1 regulates multiple genes implicated in aluminum tolerance in rice. Plant Cell. 21, 3339–3349. 10.1105/tpc.109.070771 19880795 PMC2782276

[B70] Yamamoto Y. Kobayashi Y. Matsumoto H. (2001). Lipid peroxidation is an early symptom triggered by aluminum, but not the primary cause of elongation inhibition in pea roots. Plant Physiol. 125, 199–208. 10.1104/pp.125.1.199 11154329 PMC61002

[B71] Yamamoto Y. Kobayashi Y. Devi S. R. Rikiishi S. Matsumoto H. (2003). Oxidative stress triggered by aluminum in plant roots. Plant Soil 255, 239–243. 10.1023/A:1026127803156

[B72] Yu Y. Zhou W. Zhou K. Liu W. Liang X. Chen Y. (2018). Polyamines modulate aluminum-induced oxidative stress differently by inducing or reducing H2O2 production in wheat. Chemosphere 212, 645–653. 10.1016/j.chemosphere.2018.08.133 30173111

[B73] Zhang Y. H. Wang E. M. Zhao T. F. Wang Q. Q. Chen L. J. (2018). Characteristics of Chlorophyll Fluorescence and Antioxidant-Oxidant Balance in PEPC and PPDK Transgenic Rice under Aluminum Stress. Russian J. Plant Physiol. 65, 49–56. 10.1134/s1021443718010211

[B74] Zheng X. Huystee R. B. V. (1992). Peroxidase regulated elongation of segments from peanut hypocotyls. Plant Sci. 81, 47–56. 10.1016/0168-9452(92)90023-F

[B75] Zheng S. J. Yang J. L. (2005). Target sites of aluminum phytotoxicity. Biol. Plant. 49, 321–331. 10.1007/s10535-005-0001-1

[B76] Zhu X. F. Shi Y. Z. Lei G. J. Fry S. C. Zhang B. C. Zhou Y. H. (2012). *XTH31*, encoding an in vitro XEH/XET-active enzyme, regulates aluminum sensitivity by modulating in vivo XET action, cell wall xyloglucan content, and aluminum binding capacity in. Arabidopsis Plant Cell. 24, 4731–4747. 10.1105/tpc.112.106039 23204407 PMC3531863

[B77] Zhu X. F. Wan J. X. Sun Y. Shi Y. Z. Braam J. Li G. X. (2014). Xyloglucan Endotransglucosylase-Hydrolase17 Interacts with Xyloglucan Endotransglucosylase-Hydrolase31 to Confer Xyloglucan Endotransglucosylase Action and Affect Aluminum Sensitivity in Arabidopsis. Plant Physiol. 165, 1566–1574. 10.1104/pp.114.243790 24948835 PMC4119039

