# Supplementary material for: A Maize ZmAT6 Gene Confers Aluminum Tolerance via Reactive Oxygen Species Scavenging
Source: Front Plant Sci. 2020 Jul 9;11:1016. doi: 10.3389/fpls.2020.01016 (PMC7509383; doi:10.3389/fpls.2020.01016)
Supplement: Supplementary file 1 [file Table_1.docx]

**Table S1 Basic informationof *ZmAT6***

| Gene name | Locus tag | Chr^a^ | ORF length (bp) | Protein | | | Tm domains |
| --- | --- | --- | --- | --- | --- | --- | --- |
|  |  |  |  | Length (No. of amino acids) | Mass (kDa) | pI |  |
| *ZmAT6* | GRMZM5G886177 | 3 | 771 | 256 | 27.79 | 5.8 | 0 |

^a^Chromosomal localization of *ZmAT6*
