# Supplementary material for: A Maize ZmAT6 Gene Confers Aluminum Tolerance via Reactive Oxygen Species Scavenging
Source: Front Plant Sci. 2020 Jul 9;11:1016. doi: 10.3389/fpls.2020.01016 (PMC7509383; doi:10.3389/fpls.2020.01016)
Supplement: Supplementary file 2 [file Table_2.docx]

**Table S2** *Cis*-elements in the promoter of *ZmAT6* gene

| **Factor or site name** | **Sequence** | **Function** |
| --- | --- | --- |
| A-box | CCGTCC | cis-acting regulatory element |
| ABRE | TACGTG | cis-acting element involved in the abscisic acid responsiveness |
| CAAT-box | CAAT/CAAAT | common cis-acting element in promoter and enhancer regions |
| CATT-motif | GCATTC | part of a light responsive element |
| CCGTCC-box | CCGTCC | cis-acting regulatory element related to meristem specific activation |
| CGTCA-motif | CGTCA | cis-acting regulatory element involved in the MeJA-responsiveness |
| CTAG-motif | ACTAGCAGAA |  |
| G-Box | CACGTA/TACGTG | cis-acting regulatory element involved in light responsiveness |
| GAG-motif | GGAGATG | part of a light responsive element |
| GARE-motif | TCTGTTG | gibberellin-responsive element |
| GC-motif | CCCCCG | enhancer-like element involved in anoxic specific inducibility |
| GCC box | AGCCGCC | elicitation; wounding and pathogen responsiveness |
| GCN4_motif | CAAGCCA | cis-regulatory element involved in endosperm expression |
| I-box | GATATGG | part of a light responsive element |
| GT1-motif | GGTTAA | light responsive element |
| LTR | CCGAAA | cis-acting element involved in low-temperature responsiveness |
| MBS | CAACTG | MYB binding site involved in drought-inducibility |
| P-box | CCTTTTG | gibberellin-responsive element |
| Skn-1_motif | GTCAT | cis-acting regulatory element required for endosperm expression |
| Sp1 | GGGCGG/CC(G/A)CCC | light responsive element |
| TATA-box | TTTAAAAA/TTTTA | core promoter element around -30 of transcription start |
| TGACG-motif | TGACG | cis-acting regulatory element involved in the MeJA-responsiveness |
| circadian | CAANNNNATC | cis-acting regulatory element involved in circadian control |
| SORLIP2AT | GGGCC |  |
| Unnamed__1 | CGTGG |  |
| Unnamed__4  CE3  AT-rich sequence  MSA-like  EIRE  HSE  HD-Zip 1  HD-Zip 2  plant_AP-2-like  AuxRR-core | CTCC  GACGCGTGTC  TAAAATACT  TCAAACGGT  TTCGACC  AAAAAATTTC  CAAT(A/T)ATTG  CAAT(G/C)ATTG  CGACCAGG  GGTCCAT | cis-acting element involved in ABA and VP1 responsiveness  element for maximal elicitor-mediated activation (2copies)  cis-acting element involved in cell cycle regulation  elicitor-responsive element  cis-acting element involved in heat stress responsiveness  element involved in differentiation of the palisade mesophyll cells  element involved in the control of leaf morphology development  cis-acting regulatory element involved in auxin responsiveness |

Note: High light means GGNVS site.
