# Supplementary material for: A Maize ZmAT6 Gene Confers Aluminum Tolerance via Reactive Oxygen Species Scavenging
Source: Front Plant Sci. 2020 Jul 9;11:1016. doi: 10.3389/fpls.2020.01016 (PMC7509383; doi:10.3389/fpls.2020.01016)
Supplement: Supplementary file 3 [file Table_3.docx]

**Table S3 Primers used in this study**

| **Usage of primer** | **Forward** | **Reverse** |
| --- | --- | --- |
| *ZmAT6* for amplification | 5'-GTGCAAACTAACCTTCGGATCAG-3' | 5'-CATGGACGACCAGGAACACC-3' |
| *ZmAT6* for overexpression | 5'-TATCTAGAGTGCAAACTAACCTTCGGATCAG-3' | 5'-TAGAGCTCCATGGACGACCAGGAACACC-3' |
| *ZmAT6* for subcellular localization | 5'-ATGGTACCTCGGATCAGATGGAGACGC-3' | 5'-ATGGATCCCTGTGGCTTCCCGGGGTT-3' |
| PCR detection for transgenic lines | 5'-GACCCCCACCCACGAGGAGCATC-3' | 5'-GTGCCGCCACACGGTGTGCTTC-3' |
| PCR detection for Arabidopsis mutants | 5'-TCAGGCAAAGAGTTCTTGAGC-3' | 5'-ATTTTCGACACCGTGAACAAG-3' |
|  | 5'-TGGTTCACGTAGTGGGCCATCG-3' |  |
| *ZmAT6* for RT-PCR | 5'-GTGCTCAAGGTCTACTCCGG-3' | 5'-CATCGTCCACCTTAAGCACG-3' |
| *ZmGAPDH* for RT-PCR | 5'-CCATCACTGCCACACAGAAAAC-3' | 5'-AGGAACACGGAAGGACATACCAG-3' |
| *AtAT6* for RT-PCR | 5'-CACTTAGAGCAGAGGAGATTG-3' | 5'-TTAGTGTGTGAATGGGCAGCT-3' |
| Actin for RT-PCR | 5'-GCTGGATTCTGGTGATGGT-3' | 5'-GCTCTGCTGTTGTGGTGAA-3' |
| *ZmSOD* for RT-PCR | 5'-AGTTCGGTTCTGGATGGGTT-3' | 5'-GGGTTGATGGCATTTGGAGT-3' |
| *ZmPOD* for RT-PCR | 5'-GCAGACCGCTAATCCGAA-3' | 5'-AGAAGCACCCTCCACGAA-3' |
| *ZmCAT* for RT-PCR | 5'-CAGGCTGTCGTGAGAAGTGC-3' | 5'-AGATCCAAATGGTACGGTGTTC-3' |
